# Achieving Efficiency in Full Endoscopic Interlaminar Lumbar Decompression: A Prospective Learning-Curve Cumulative Summation Analysis and Its Impact on Patient-Reported Outcomes

**DOI:** 10.7759/cureus.98634

**Published:** 2025-12-07

**Authors:** José Machado, Patrícia Martins, Pedro Mendes Santos, Ricardo Vila Real, José Costa, Leonor Rocha, António Neto, Nuno Bastos

**Affiliations:** 1 Orthopaedics, Hospital Pedro Hispano, Matosinhos, PRT

**Keywords:** full-endoscopic spine surgery, interlaminar approach, lc‑cusum, lumbar spinal stenosis, minimally-invasive spine surgery, patient-reported outcomes, prospective cohort, surgical learning curve

## Abstract

Background and objective

Full endoscopic interlaminar lumbar decompression is increasingly being adopted for lumbar spinal stenosis, yet the learning curve and its clinical consequences remain uncertain. In this study, we aimed to prospectively evaluate a single surgeon’s learning curve for full endoscopic interlaminar decompression.

Methods

We conducted a prospective, single-surgeon cohort (2018-2023) involving adults who underwent single-level full endoscopic interlaminar decompression for degenerative lumbar canal stenosis. Operative efficiency was prespecified as operative time ≤90 minutes. Learning-curve cumulative summation (LC-CUSUM) was applied to detect the transition from inadequate to adequate performance; competency was declared at the first boundary crossing. Patient-reported outcomes included the Oswestry Disability Index (ODI) and Visual Analog Scale (VAS) scores collected at baseline and at one, three, six, and 12 months. Safety endpoints included major complications and reoperations.

Results

A total of 77 patients were included (mean follow-up period: 47 months). LC-CUSUM signaled efficiency competency at case 44. Mean operative time was 98.6 ± 29.0 minutes overall; 111.2 ± 32.0 minutes in the early phase group (cases 1-44) and 81.7 ± 10.1 minutes in the late phase group (cases 45-77). Both groups demonstrated significant within-group improvements in ODI and VAS at each postoperative time point; however, the magnitude of improvement did not differ between phases. Major complications (1/44 early vs. 0/33 late) and reoperations (3/44 vs 2/33) were comparable. All patients were discharged within 24 hours.

Conclusions

Efficiency competency was achieved without compromising patient‑reported outcomes or safety across the learning curve.

## Introduction

Degenerative lumbar spinal stenosis is a leading indication for decompression in older adults and is characterized clinically by neurogenic claudication and radicular or mixed back/leg pain related to progressive ligamentum flavum hypertrophy, facet overgrowth, capsular thickening, and disc degeneration [1-3]. Functional limitation in this population often justifies operative decompression when conservative care is inadequate.

Spine surgery outcomes depend not only on indication and technique but also on intraoperative execution and surgeon experience [4]. Full endoscopic lumbar decompression through an interlaminar approach has gained traction as a less invasive alternative to conventional microscopic or tubular decompression, aiming to minimize muscular detachment and bony resection while achieving adequate neural decompression. Randomized and comparative studies report that full endoscopic techniques offer non-inferior effectiveness with faster recovery times in lumbar stenosis [5,6], while meta-analytic evidence suggests comparable or lower complication rates with similar or greater leg-pain improvement compared with conventional techniques [6,7].

Because endoscopic spine surgery demands precise endoscope handling, continuous irrigation control, and decompression through a narrow working corridor, structured evaluation of the learning curve is essential for safe adoption. Learning-curve cumulative summation (LC-CUSUM) inverts the classical CUSUM hypothesis framework to detect the transition from inadequate to adequate performance using predefined error thresholds and performance benchmarks [8]. This approach enables case-by-case monitoring of whether a surgeon has achieved an acceptable and stable level of performance, rather than relying on an arbitrary case volume to assume competency.

In this study, we prospectively evaluate a single surgeon’s learning curve for full endoscopic interlaminar decompression. Our primary aim is to use LC-CUSUM to identify the case at which operative efficiency competency is achieved during the adoption of full endoscopic interlaminar decompression. Our secondary objectives are to compare patient-reported outcomes, major complications, and reoperations before and after the established competency point.

## Materials and methods

Study design and patients

We conducted a prospective, single-surgeon cohort study at a tertiary hospital between 2018 and 2023. Consecutive adults with degenerative lumbar canal stenosis undergoing single-level full endoscopic interlaminar decompression were enrolled. Cases were enrolled consecutively and indexed chronologically; no training interruptions occurred during the series. Exclusion criteria included prior lumbar surgery; trauma, tumor, or infection; radiographic instability; degenerative spondylolisthesis beyond Meyerding grade I; scoliosis greater than 20°; multilevel decompression; or follow-up of less than 18 months. The learning-curve analysis determined the point of efficiency competency, after which the cohort was divided into an early-phase group (all cases up to and including the competency case) and a late-phase group (all subsequent cases).

Technical note: surgical technique

All procedures were performed using a standard interlaminar full endoscopic approach with the joimax®iLESSYS® or iLESSYS® Pro systems (Karlsruhe, Germany). General anesthesia was administered without neuromuscular blockade, and patients were positioned prone on a radiolucent table. All patients received standard single-dose cefazolin (or clindamycin and gentamicin if allergic). Under fluoroscopic guidance, a small paramedian incision was created; serial dilation was used to dock a working cannula onto the interlaminar window. An endoscope with an integrated working channel was introduced, and decompression proceeded under continuous irrigation. A limited laminotomy and flavectomy were carried out to decompress the ipsilateral lateral recess; when indicated, contralateral recess and central decompression were achieved by undercutting the spinous process base. Hemostasis was obtained using radiofrequency and endoscopic instruments. The skin was closed with two simple interrupted sutures, and no drain was used. All patients began immediate postoperative mobilization, and no postoperative venous thromboembolism prophylaxis was used. Discharge criteria included independent ambulation, adequate oral analgesia, and spontaneous voiding

Efficiency endpoint and LC-CUSUM

Operative time (skin incision to wound closure) was the prespecified efficiency endpoint. A case was considered a success if the operative time was at or below 90 minutes and a failure if greater than 90 minutes. The 90-minute success threshold was set a priori by departmental consensus as a pragmatic efficiency goal aligned with our service benchmark for single-level decompression and allowing for endoscopy-specific setup time. We defined a binary indicator for each case and modeled the underlying failure probability. \( X_i= \begin{cases} 1, & \text{failure (operative time } > 90 \text{ min)}\\ 0, & \text{success (operative time } \le 90 \text{ min)} \end{cases}\)

LC-CUSUM inverts the classical CUSUM hypotheses to detect the transition from inadequate to adequate performance. 

\(H_0:\ p=p_0 \quad \text{(out-of-control, inadequate)}, \qquad H_1:\ p=p_1 \quad \text{(in-control, adequate)}\)

The parameters were set a priori as unacceptable failure rate p_0_, acceptable failure rate p_1_, type I error α, and type II error β, in line with prior learning-curve-related literature. 

$p_0=0.40,\quad p_1=0.20,\quad \alpha=0.05,\quad \beta=0.20$ For each case, LC-CUSUM adds a log-likelihood increment that depends on success or failure.

\(w_i= \begin{cases} \log\!\left(\dfrac{p_0}{p_1}\right), & X_i=1\ \text{(failure)},\\[6pt] \log\!\left(\dfrac{1-p_0}{1-p_1}\right), & X_i=0\ \text{(success)} \end{cases}\) The cumulative score is updated with a holding barrier at zero to avoid banking of early credit. 

\(S_0=0, \qquad S_k=\min\!\bigl(0,\ S_{k-1}+w_k\bigr), \quad k\ge 1\)

A one-sided decision limit $h=log⁡ ⁣((1&minus;&beta;)/&alpha;)$ was used; competency was declared at the first case $k$ such that $Sk​&le;&minus;h$

Using natural logarithms with the chosen parameters, the numeric values are: 

\(\log\!\left(\dfrac{1-p_0}{1-p_1}\right) =\log\!\left(\dfrac{0.60}{0.80}\right) =\log(0.75)\approx -0.288\) \(\log\!\left(\dfrac{p_0}{p_1}\right) =\log\!\left(\dfrac{0.40}{0.20}\right) =\log(2)\approx +0.693\) \(h=\log\!\left(\dfrac{1-\beta}{\alpha}\right) =\log\!\left(\dfrac{0.80}{0.05}\right) =\log(16)\approx 2.773\)

After the LC-CUSUM competency signal, performance can be tracked with a conventional (two-sided) CUSUM for deterioration using a symmetric decision interval.

Outcomes and statistical analysis

Patient-reported outcomes included the Oswestry Disability Index (ODI) and the Visual Analog Scale (VAS) for pain, collected at baseline and at one, three, six, and 12 months. Use of the ODI complied with academic research terms; the instrument and licensing resource are cited [9,10]. The VAS was used according to the original description [11]. Continuous variables are summarized as mean ± standard deviation (SD) and categorical variables as n (%). Major complications were defined a priori as events requiring conversion to open surgery, dural tear, new neurologic deficit, deep infection, or other unplanned invasive intervention.

Within each phase, change from baseline was tested with paired t-tests and is reported with the t statistic and degrees of freedom (df). Between-phase differences in change (late minus early) were evaluated with Welch's t-tests using Satterthwaite df; we report the mean difference, the 95% confidence interval (CI), the t statistic with df, and the p-value. Binary outcomes (major complications and reoperations) were compared with Fisher's exact test and expressed as odds ratios (OR) with 95% CIs. A two-sided p < 0.05 was considered statistically significant.

Time measurements for each operative step were summarized and analyzed with SPSS Statistics, version 27.0 (IBM Corp., Armonk, NY). LC-CUSUM calculations and figure rendering were implemented in Python.

## Results

**Figure 1:**
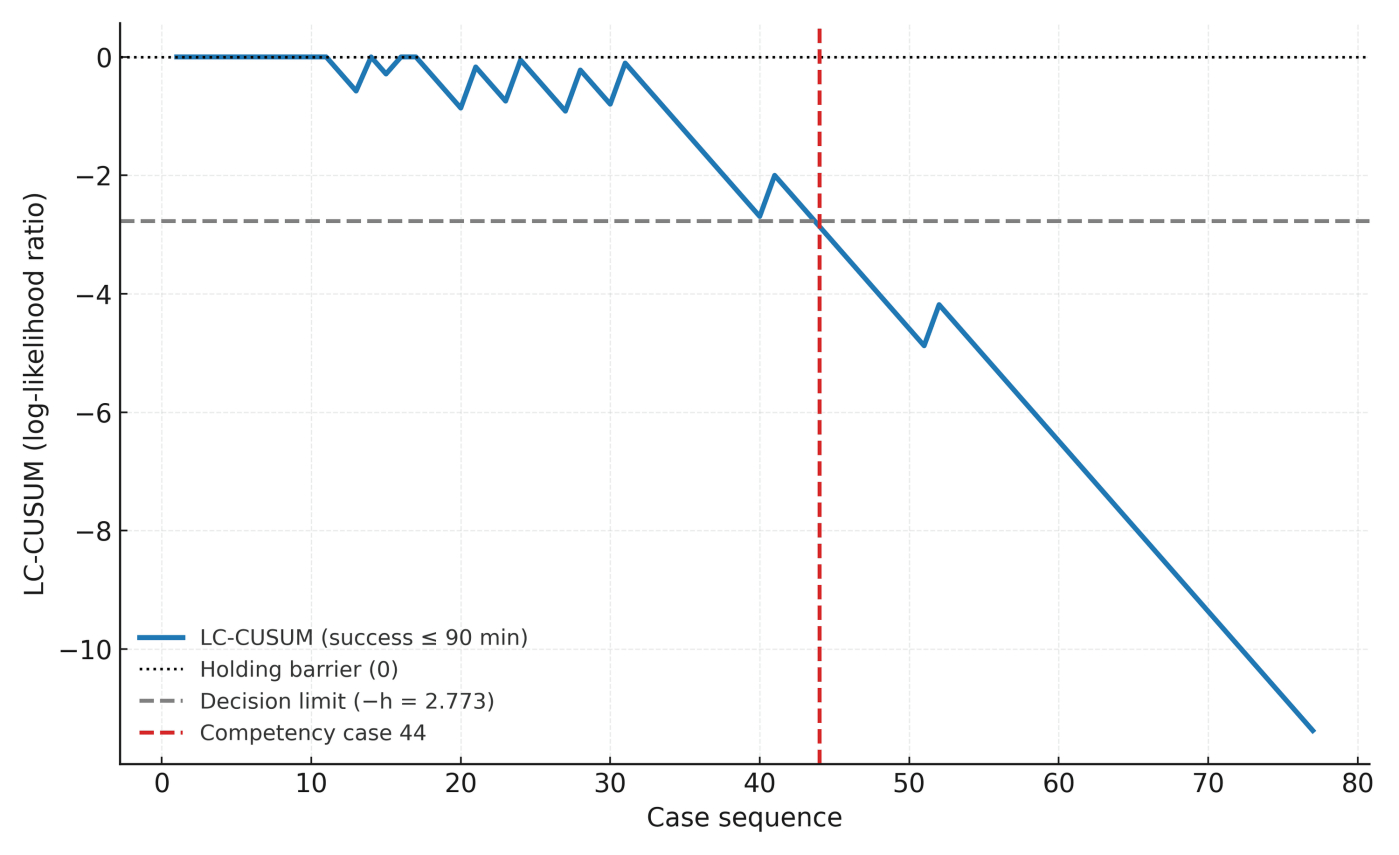
LC‑CUSUM for operative time (success ≤90 minutes) LC‑CUSUM: learning-curve cumulative summation

A total of 77 patients were included in the analysis. The mean follow-up duration was 47 months. Using LC-CUSUM with a success threshold defined as an operative time of ≤90 minutes, the initial point of competency was reached at case 44 (Figure 1). Accordingly, cases 1-44 were classified as the early-phase group (n = 44), and cases 45-77 as the late-phase group (n = 33). The mean operative time was 98.6 ± 29.0 minutes overall; 111.2 ± 32.0 minutes in the early-phase group versus 81.7 ± 10.1 minutes in the late-phase group. Operative time was shorter in the late-phase group by 29.5 minutes, and this difference was statistically significant (Welch t = −5.74, df = 54, p < 0.0001), indicating marked improvement in efficiency after competency was achieved.

The groups did not differ significantly in age, sex, BMI, or comorbidities, indicating baseline homogeneity. All patients were discharged within 24 hours.

ODI and VAS improved from baseline at one, three, six, and 12 months in both groups (Table 1, Table 2); however, between-group differences in improvement were not statistically significant at any time point (all p > 0.05) (Table 3, Table 4).

**Table 1:** Mean ODI at baseline and follow‑up ODI: Oswestry Disability Index; SD: standard deviation

Timepoint	Early, n	Early, mean ± SD	Early, Δ, mean ± SD	Early, t	Early, df	Early, p (paired)	Early, Cohen dz	Late, n	Late, mean ± SD	Late, Δ, mean ± SD	Late, t	Late, df	Late, p (paired)	Late, Cohen dz
ODI baseline (0)	44	0.296 ± 0.130	—	—	—	—	—	33	0.285 ± 0.101	—	—	—	—	—
ODI 1 month	44	0.137 ± 0.123	-0.159 ± 0.104	-10.14	43	<0.0001	-1.53	33	0.119 ± 0.076	-0.167 ± 0.095	-10.10	32	<0.0001	-1.75
ODI 3 months	44	0.112 ± 0.105	-0.184 ± 0.106	-11.52	43	<0.0001	-1.74	33	0.101 ± 0.072	-0.184 ± 0.106	-9.97	32	<0.0001	-1.75
ODI 6 months	44	0.104 ± 0.100	-0.192 ± 0.114	-11.18	43	<0.0001	-1.68	33	0.098 ± 0.085	-0.187 ± 0.118	-9.10	32	<0.0001	-1.59
ODI 12 months	44	0.123 ± 0.113	-0.174 ± 0.126	-9.16	43	<0.0001	-1.38	33	0.085 ± 0.086	-0.201 ± 0.117	-9.87	32	<0.0001	-1.72

**Table 2:** Mean VAS at baseline and follow-up VAS: Visual Analog Scale; SD: standard deviation

Timepoint	Early, n	Early, mean ± SD	Early, Δ, mean ± SD	Early, t	Early, df	Early, p (paired)	Early, Cohen dz	Late, n	Late, mean ± SD	Late, Δ, mean ± SD	Late, t	Late, df	Late, p (paired)	Late, Cohen dz
VAS baseline (0)	44	7.727 ± 1.909	—	—	—	—	—	33	7.727 ± 1.892	—	—	—	—	—
VAS 1 month	44	2.636 ± 2.488	-5.091 ± 2.995	-11.28	43	<0.0001	-1.70	33	3.273 ± 2.169	-4.455 ± 2.762	-9.26	32	<0.0001	-1.61
VAS 3 months	44	2.886 ± 2.517	-4.841 ± 2.710	-11.86	43	<0.0001	-1.79	33	2.424 ± 2.488	-5.303 ± 3.057	-9.96	32	<0.0001	-1.73
VAS 6 months	44	2.705 ± 2.436	-5.023 ± 2.774	-12.01	43	<0.0001	-1.81	33	2.576 ± 2.598	-5.152 ± 3.163	-9.35	32	<0.0001	-1.63
VAS 12 months	44	3.136 ± 2.858	-4.591 ± 3.336	-9.13	43	<0.0001	-1.38	33	2.758 ± 2.646	-4.970 ± 3.177	-8.98	32	<0.0001	-1.56

**Table 3:** ODI change (early-phase group vs. late-phase group) ODI: Oswestry Disability Index; SD: standard deviation; CI: confidence interval

Interval, months	Early, n	Early, Δ, mean ± SD	Late, n	Late, Δ, mean ± SD	Δ (late-early)	95% CI, low	95% CI, high	Welch t	Welch df	P-value
0→1	44	-0.159 ± 0.104	33	-0.167 ± 0.095	-0.007	-0.053	0.038	-0.31	72	0.7481
0→3	44	-0.184 ± 0.106	33	-0.184 ± 0.106	0.000	-0.049	0.049	0.00	69	0.9975
0→6	44	-0.192 ± 0.114	33	-0.187 ± 0.118	0.005	-0.048	0.058	0.18	68	0.8524
0→12	44	-0.174 ± 0.126	33	-0.201 ± 0.117	-0.027	-0.082	0.029	-0.95	72	0.3358

**Table 4:** VAS change (early-phase group vs. late-phase group) VAS: Visual Analog Scale; SD: standard deviation; CI: confidence interval

Interval, months	Early, n	Early, Δ, mean ± SD	Late, n	Late, Δ, mean ± SD	Δ (late-early)	95% CI, low	95% CI, high	Welch t	Welch df	P-value
0→1	44	-5.091 ± 2.995	33	-4.455 ± 2.762	0.636	-0.679	1.951	0.95	72	0.3379
0→3	44	-4.841 ± 2.710	33	-5.303 ± 3.057	-0.462	-1.802	0.878	-0.68	64	0.4934
0→6	44	-5.023 ± 2.774	33	-5.152 ± 3.163	-0.129	-1.510	1.253	-0.18	64	0.8528
0→12	44	-4.591 ± 3.336	33	-4.970 ± 3.177	-0.379	-1.869	1.112	-0.50	71	0.6139

Major complications consisted of one intraoperative dural tear requiring conversion to open surgery in the early-phase group; no other major events met our predefined criteria. Rates of major complications and reoperations were similar between the two groups (Table 5).

**Table 5:** Complications and reoperations (early-phase group vs. late-phase group) OR: odds ratio; CI: confidence interval

Outcome	Early, n (%)	Late, n (%)	OR (late vs. early)	95% CI, low	95% CI, high	Test	Test statistic	P-value
Major complication	1/44 (2.3%)	0/33 (0.0%)	0.33	0.01	8.10	Fisher's exact	Statistic: N/A	1.000
Reoperation	3/44 (6.8%)	2/33 (6.1%)	0.93	0.15	5.65	Fisher's exact	Statistic: N/A	1.000

## Discussion

This prospective study shows that efficiency competency in full endoscopic interlaminar decompression was achieved partway through a surgeon’s initial series, as determined by predefined LC-CUSUM criteria, a validated approach for monitoring learning-curve transitions in procedural performance [8,12]. Notably, the magnitude of ODI and VAS improvement up to 12 months was comparable before and after competency, and adverse-event and reoperation rates did not differ. These findings indicate that efficiency gains primarily reflect technical fluency and workflow optimization rather than a change in decompression adequacy or overall clinical benefit.

Our operative-time trajectory and stable safety profile align with prior full endoscopic and unilateral biportal endoscopy learning-curve studies employing CUSUM/LC-CUSUM across spine procedures [13-19]. Comparative and randomized evidence suggests that, once technically mature, full endoscopic decompression yields outcomes comparable to microscopic/tubular techniques, with faster recovery and low perioperative morbidity, including shorter length of stay and reduced blood loss in randomized trials and meta-analyses [5-7]. Universal discharge within 24 hours in our series supports the feasibility of short-stay pathways after endoscopic decompression.

Strengths and limitations

The strengths of this study include its prospective design, homogeneous single-level indication, explicit a priori LC-CUSUM criteria with a holding barrier, standardized follow-up to 12 months, and a mean follow-up of 47 months, ensuring mature outcome capture. However, the study has certain limitations too: limited generalizability due to the single-surgeon setting, potential secular trends in case mix, reliance on operative time as a single efficiency endpoint rather than a composite, absence of risk-adjusted CUSUM, possible case-mix heterogeneity across the study period, and the influence of team learning curve. Future multicenter studies should incorporate risk-adjusted LC-CUSUM and multidimensional performance metrics (e.g., blood loss, same-day discharge, readmission) and explore curriculum development informed by learning-curve analytics.

## Conclusions

Using a ≤90-minute operative time threshold, LC-CUSUM indicated that efficiency competency was reached at case 44. Despite significantly shorter operative times after competency, ODI and VAS improvements, major complications, reoperations, and length of stay were comparable across phases. Overall, our results suggest that full endoscopic interlaminar lumbar decompression can be safely adopted during the learning phase without diminishing patient-reported outcomes, and that growing experience chiefly improves operative efficiency rather than clinical benefit.

## Appendices

# LC-CUSUM minimal reproducible code (Python 3) import math def lc_cusum(binary_fail_list, p0=0.40, p1=0.20, alpha=0.05, beta=0.20): w_fail = math.log(p0/p1) w_succ = math.log((1-p0)/(1-p1)) h = math.log((1-beta)/alpha) s = 0.0 series = [] for x in binary_fail_list: w = w_fail if x==1 else w_succ s = min(0.0, s + w) series.append(s) return series, h
 
